# Synergy Effect of Combined Near and Mid-Infrared Fibre Spectroscopy for Diagnostics of Abdominal Cancer

**DOI:** 10.3390/s20226706

**Published:** 2020-11-23

**Authors:** Thaddäus Hocotz, Olga Bibikova, Valeria Belikova, Andrey Bogomolov, Iskander Usenov, Lukasz Pieszczek, Tatiana Sakharova, Olaf Minet, Elena Feliksberger, Viacheslav Artyushenko, Beate Rau, Urszula Zabarylo

**Affiliations:** 1 Department of Surgery, Charité University of Berlin, 13353 Berlin, Germany; thocotz@gmail.com (T.H.); beate.rau@charite.de (B.R.); 2 Art Photonics GmbH, Rudower Chaussee 46, 12489 Berlin, Germany; iu@artphotonics.de (I.U.); ts@artphotonics.de (T.S.); ef@artphotonics.de (E.F.); sa@artphotonics.de (V.A.); 3 Laboratory of Multivariate Analysis and Global Modelling, Samara State Technical University, Molodogvardeyskaya 244, 443100 Samara, Russia; valerya.belickova@yandex.ru (V.B.); ab@globalmodelling.com (A.B.); 4 Institute of Optics and Atomic Physics, Technical University of Berlin, 10623 Berlin, Germany; 5 Institute of Chemistry, University of Silesia in Katowice, 9 Szkolna Street, 40006 Katowice, Poland; lukasz.pieszczek@us.edu.pl; 6 CC06-CharitéCentrum 6, Center for Diagnostic and Interventional Radiology and Nuclear Medicine, Charité Universitätsmedizin Berlin, CBF Campus Benjamin Franklin, Hindenburgdamm 30, 12203 Berlin, Germany; olaf.minet@charite.de; 7 BIH Berlin Institute of Health, BCRT-Center for Regenerative Therapies, Cranach Haus, Charité - Universitätsmedizin Berlin, CVK Campus Virchow-Klinikum, Augustenburger Platz 1, 13353 Berlin, Germany; urszula.zabarylo@charite.de

**Keywords:** mid-infrared spectroscopy, near-infrared spectroscopy, FTIR absorbance, diffuse reflection, synergy effect, fibre probe, joint data analysis, cancer diagnostics

## Abstract

Cancers of the abdominal cavity comprise one of the most prevalent forms of cancers, with the highest contribution from colon and rectal cancers (12% of the human population), followed by stomach cancers (4%). Surgery, as the preferred choice of treatment, includes the selection of adequate resection margins to avoid local recurrences due to minimal residual disease. The presence of functionally vital structures can complicate the choice of resection margins. Spectral analysis of tissue samples in combination with chemometric models constitutes a promising approach for more efficient and precise tumour margin identification. Additionally, this technique provides a real-time tumour identification approach not only for intraoperative application but also during endoscopic diagnosis of tumours in hollow organs. The combination of near-infrared and mid-infrared spectroscopy has advantages compared to individual methods for the clinical implementation of this technique as a diagnostic tool.

## 1. Introduction

Abdominal cancers comprise of several cancer types between the lower chest and the groin, which include the stomach, small intestines, colon, liver, gallbladder, pancreas, spleen, kidneys, and adrenal glands. They are one of the most prevalent forms of cancer, with the highest contribution from colorectal cancers (12% of the human population), followed by stomach cancer (4%) [1]. The 5-year survival varies from more than 50% for colorectoral cancer to less than 8% for pancreatic cancer [2].

Surgery, as one of the preferred treatment modalities, aims to remove cancerous tissue, including a margin of healthy tissue, making the selection of resection margins as one of the foremost challenges for a surgeon [3]. Inadequate resection margins may lead to the increased risk of local recurrence or the need for additional resection [4], while the severe side effects of radical surgery lead to a considerable deterioration of patient health and quality of life [5]. As a result, new diagnostic methods are being sought to accurately and rapidly assess the extent of tumour margins and to distinguish malignant tissues from normal ones more efficiently.

Optical spectroscopy methods offer unique opportunities to investigate the sample properties at the molecular level. Since every chemical species in a sample has a unique spectrum, the overall chemical composition can be evaluated through the spectral analysis by using sophisticated methods of modern data analysis, which are referred to as chemometrics [6]. In that context, on/in-line spectroscopy has proven to be a valuable and efficient analytical tool for medical diagnostics and general tissue studies in biology and medicine [7,8]. As a new diagnostic tool, it offers unique opportunities for a label-free investigation of tissue samples at the molecular level, serving as an ancillary instrument for classical histopathology, and it is sometimes referred to as “spectral histopathology” [9,10]. Optical fibres play a vital role in the translation of spectroscopy methods into clinical and preclinical practice: the ability to miniaturise fibres, their considerable flexibility, and their ease of use facilitate the translation of such devices to the clinical setting. This technology would allow in situ analysis in real time with a minimal requirement of optical alignment and maximum safety [11]. Near-infrared (NIR) fibre-optic spectroscopy is one of the most established spectroscopic methods that is successfully used in measuring water content in the skin [12], brain oxygenation in stroke patients [13], and in cancer research [14,15]. Near-infrared spectra are formed by combinations and overtones of fundamental vibrations of C–H, N–H, O-H, and other functional groups [14]. NIR spectroscopy provides information on different biologically important molecules (e.g., proteins, lipids, glucose, collagen, globulins) [16] and is capable of diagnosing several diseases [16], including cancer of various organs [17,18]. However, the overlap of individual absorptions, resulting in broad, unspecific bands limits the ability to identify concentrations of specific molecular species contained in the sample [16,19]. Vibrational spectroscopy is one of the fundamental physical methods of chemical analysis that avoids the limitation of low molecular recognition accuracy caused by the broadness of spectral bands in NIR reflection [20]. Molecular-specific vibrational absorptions in the mid-infrared (MIR) fingerprint region, which are characteristic for various anti-symmetric vibrations (e.g., for polar bonds such as O–H or N–H), enable the unambiguous molecular identification of many major and minor compounds present in the samples [21]. Despite classical MIR transmission spectra of biological tissue being rather complex to analyse, there have been many successful attempts to apply attenuated total reflection (ATR) MIR spectroscopy to various types of human cancer [22,23,24] to evaluate early-stage cartilage degradation [25] and for the analysis of sediments in various body fluids [26]. Moreover, there are proof-of-concept examples of clinical applications of MIR spectroscopy in Fourier transform mode (FT-IR) that are used for the enhancement of histopathology and cytology [27]. FTIR can be applied not only for cancer diagnosis but also for the interrogation of different tissues to diagnose diabetic nephropathy progression [28], systemic sclerosis [29], or systemic amyloidosis [30].

A combination of several spectroscopic modalities enables us to reveal information that was previously not accessible and eliminates the drawbacks of individual approaches. In the last decade, it was demonstrated that a combination of fibre-optic approaches with the fusion of spectroscopic data from several methods increases the accuracy of sample differentiation by tens of percent [31,32]. Recently, authors demonstrated that a combination of a fibre-optic approach with the fusion of the spectroscopy data from several methods also increases the accuracy of tumour detection by 28% [33]. However, the majority of reported multispectral analyses present a combination of fluorescence and NIR spectroscopy methods [32,34]. Here, we present a fusion of datasets obtained from two complementary techniques enclosing the information about the molecular differences on the cellular level (ATR MIR absorption) and the changes in tissue properties at a significant penetration depth of several millimeters (NIR reflection). The main advantage of MIR analysis is the ability to precisely interpret analytical information provided by spectral signatures of functional groups present in its molecular structure (direct spectral information). The latter enables the detection of different cancer types based on the qualitative and quantitative correlation of specific cancer biomarkers. MIR radiation penetrates the sample by up to 2 μm, which allows analysing individual cellular structures [33], while NIR radiation penetrates up to several mm into the tissue, allowing the evaluation of several tissue layers (e.g., epithelial cells, mucosa, muscular) [14]. We assume that this ability could vastly enhance the information collection process and become an essential part of the clinical translation of a new cancer diagnostic tool.

For a dataset combining several heterogeneous parts, two strategies of data analysis could be considered: (i) to apply one common universal model covering the entire dataset and (ii) to divide the samples into groups and consider the corresponding datasets separately. It is necessary to find the optimal division of the dataset to determine which approach would provide the most accurate solution. The current results represent a part of a more comprehensive research project with the primary aim to develop new optical techniques for tumour margin identification and non-invasive cancer diagnosis in real time, including the translation of the technique into the clinical environment as a supportive diagnostic tool. The authors’ recent work from this series was aimed at the development and testing of a NIR sensor for the diagnosis of kidney tumours based on light-emitting diodes (LEDs) [15]. Furthermore, the synergy effect of the concomitant MIR and fluorescence spectroscopy use for analysis of kidney tumours [33] or NIR and fluorescence spectroscopy for analysis of colon tumours was demonstrated [35]. In this study, we applied an equivalent approach. The novelty lies in testing an entirely different combination of near- and mid-infrared fibre-optic spectroscopy techniques to detect several abdominal cancers, i.e., stomach, colon, and rectal cancer, and to demonstrate a similar synergy effect of their combination. Exceeding the scope of previous publications, not only the separation of normal and cancerous tissue samples of the same organ but also the spectroscopic separation between the different organ types was intended.

## 2. Materials and Methods

### 2.1. Sample Collection

Altogether, 70 unstained tissue samples were collected from 35 patients suffering from colorectal or stomach cancer. From each patient, two correlating ex vivo samples (one sample of cancerous tissue and one sample of healthy tissue) were taken. The samples were acquired during the planned surgery for open or laparoscopic resection of cancerous tissue. Informed patient consent was acquired at least 24 h before the surgery. The tissue sampling and further investigation of biopsies were approved by the institutional ethics committee (ethical approval number EA1/159/16). All samples were collected at two independent departments of general surgery of the Charité—Universitätsmedizin Berlin, Germany—Campus Charité Mitte (CCM) and Virchow—Klinikum (CVK).

The resected tissue was transported in a sealed container to the pathology department within 30 min of resection. From each resectate, a trained pathologist took similar samples of cancerous and normal tissue (5–10 mm thickness) with appropriate distance (at least 5 cm) from each other. The specimens were mounted on cork tiles, fixed with pins, and immediately quick-frozen in liquid nitrogen. Samples were stored and transported exclusively at −80 °C until the spectroscopic measurement.

### 2.2. Sample Preparation

A 37 °C water bath without direct water contact was used to thaw the samples. The samples were subsequently fixed in a petri-dish, in the same orientation as on the cork tiles using tissue glue (TRUGLUE^®^, Trusetal, Germany) to avoid sample displacement during measurement. The Petri dish was equipped with a colour- and position-coded (blue = normal; red = cancerous tissue) 3 mm × 3 mm grid for measurement point determination. The surface of the tissue was washed with saline (NaCl 0.9%). For each specimen, at least three distinct points were selected. Mid-infrared absorption (MIR) and diffuse reflection (NIR) measurements were performed using optical fibre probes placed in slight contact with the tissue surface. During each measurement, the probe was placed in contact with the sample manually. Through this process, we intended to cover the entire outer silica crystal tip with the examined tissue surface. Other specific measurement procedures that ensure a constant contact pressure between the probe and the tissue samples were not implemented during this study. The objective of the applied experimental design was to mimic in vivo conditions during endoscopic examinations of the gastrointestinal tract. The accurate spatial coincidence of measurements with two independent spectroscopic methods was ensured by performing the measurements in preselected positions following the position code marked on the grid. Three measurements were performed using each spectroscopic method in each preselected position. Additional specifications about the sample preparation can be found in previous publications by the authors [15,33].

### 2.3. Spectroscopic Measurements

A Matrix MF (Bruker Optik GmbH, Ettlingen, Germany) spectrometer was employed for MIR spectroscopic measurements. It was equipped with a mercury–cadmium–telluride (MCT) detector cooled by liquid nitrogen. A polycrystalline infrared (PIR) fibre-based attenuated total reflection (ATR) probe topped with a silica crystal (art photonics GmbH, Berlin, Germany) was used for the acquisition of spectra. The probe had been adjusted for measurements of tissue samples in the spectroscopic fingerprint region (1800 to 900 cm^−1^). In such a range, MIR spectra of studied samples were acquired with the spectral resolution of 8 cm^−1^. Each collected spectra was the result of 64 averaged scans. A sterile 0.9% sodium chloride physiological solution was used as a reference sample for spectra calibration.

Spectra in the NIR range were acquired using a portable fibre-optic NIRQuest512 spectrometer (Ocean Optics, Inc., Orlando, FL, USA) equipped with an indium gallium arsenide detector. The source of light used during the measurements was a LS-1 tungsten halogen lamp (Ocean Optics, Inc., Orlando, FL, USA). The spectrometer was supplied with a fibre-optic probe with eight 400 mm fibres—one for light emission and seven for light collection (art photonics GmbH, Berlin, Germany). The recorded spectral NIR range was from 900 to 1700 nm, and the spectral resolution was equal to 1.66 nm. The exposure time of the measurements and number of scans collected for spectra averaging were 150 ms and 5, respectively. Measurements of a white reference material—Spectralon (Labsphere, Inc, North Sutton, NH, USA) and closed spectrometer slit (dark reference) were conducted to calibrate the spectral intensity of the sample spectra. The spectral intensity calibration was performed with collected white and dark references through so called unity-based normalisation (UBN) approach [36].

### 2.4. Histopathological Evaluation

To confirm the presence or absence of cancer cells in the measured samples, the spectroscopically inspected surface of the specimens underwent histopathological evaluation via a standardised frozen thin-section technique. Only samples with a confirmed presence or absence of cancer cells were used for chemometric modelling.

The detailed description of the samples is presented in Table 1 that shows the amount of cancer tissue samples in which cancer tissue could be confirmed. The reduced number of confirmed cancer samples in the stomach group can be explained by the comprehensive application of neoadjuvant chemotherapy, completely eradicating the cancerous tissue in over 50% of cases. The reduced number of confirmed malignant samples in the stomach cancer group was caused by the presence of diffuse stomach cancer subtype in 4(N)/19(N) patients. Only spectroscopic data of confirmed cancer and normal samples was utilised for the data analysis and modelling aspects of the study.

### 2.5. Data Analysis

Each tissue sample was measured three to five times in three to five positions, depending on the size of the sample. Repeated measurements were averaged. Each row in a dataset matrix (i.e., position) corresponds to a unique position on one of the tissue samples, and several subsequent rows (i.e., positions) in the matrix correspond to one individual patient (tumour and normal tissue samples).

In some cases, the size and consistency of the biopsy were not suitable for complete measurement with both spectroscopic methods. Therefore, the original, full MIR dataset, including colon samples of 10 patients resulted in 73 different positions, 15 stomach samples resulted in 93 positions, and 6 rectum samples resulted in 37 positions. The original, full NIR dataset, including colon samples of 10 patients resulted in 54 different positions, 13 stomach samples resulted in 61 positions, and 6 rectum samples resulted in 28 positions. The main difficulty in testing the synergy effect is the necessity of using the same position measured by both spectroscopic methods. Since this was not always possible, there are only 52 colon sample positions (27N, 25T) from 10 patients, 52 stomach sample positions (32N, 20T) from 12 patients, and 21 rectum sample positions (9N, 12T) from 6 patients that were used in this work. Reduced datasets were used also for the construction of individual models based on one spectroscopic technique (NIR or MIR) to reliably compare the diagnostic potential of concatenated data. The reason behind such an idea was to avoid the influence of imbalanced quality and size of different datasets, which itself could be a strong source of additional variance and may hamper the final interpretation and comparison of the diagnostic performance of investigated approaches.

Multivariate data analysis and data visualisation were performed with TPT-cloud (www.tptcloud.com, Global Modelling, Aalen, Germany and Samara State Technical University, Samara, Russia) and Interval Selection Toolbox for Matlab^TM^ (MathWorks, Natick, MA, USA). Before the analysis was conducted, repeated measurements were averaged. Partial least-squares discriminant analysis (PLS-DA) [37] was used for building classification models. Moreover, we applied Variable Importance in Projection (VIP) method. The exact methodology of VIP is described elsewhere [38]. Leave-one-out (LOO) [39,40] and Monte Carlo [41,42] cross-validation were used to test the model performance. During Monte Carlo cross-validation, 10% of the complete dataset was removed in an iterative procedure. The validation cycle was repeated 100 times for each model. Raw datasets and datasets pretreated by different methods [43] were considered, including standard normal variate correction (SNV), first (1D) and second (2D) derivative (by Savitzky–Golay algorithm with a second-order polynomial and window width of 25 points for NIR data and 8 points for MIR data), first and second derivative and SNV (derivative followed by SNV). Additional normalisation (max intensity is 1, and min intensity is 0) was used for preprocessed NIR and MIR spectra to concatenate both parts correctly. It is important to note that for each data part (for each spectroscopic method), the whole series of the spectra were scaled with the same coefficient. In the present paper, we have decided to use this normalisation strategy to avoid any further increase of complexity during data analysis and to ensure the same contribution of NIR and MIR data parts in models based on concatenated spectroscopic data.

NIR data in the figures were also smoothed by Savitzky–Golay filter (with second-order of polynomial and window width of 25 points).

In PLS-DA modelling, 1 (“positive” test results) indicates designated tumours, and 0 (“negative” test results) indicates designated normal samples. Sensitivity, specificity, and accuracy statistics were used for a calibration dataset and cross-validation prediction to measure discrimination quality. Additional specifications can be found in previous publications by the authors [33].

### 2.6. Models Ranking Using ROC-Curves

A receiver operating characteristic curve (ROC-curve) was used for the model comparison, which was created by plotting the true positive rate, i.e., sensitivity (Sn) against the false positive rate, i.e., reversed specificity (1-Sp) at various threshold settings [44]. In other words, each PLS-DA model is represented by a curve in the ROC-space, and each point on the ROC-curve corresponds to a model statistics for a certain threshold. One point in the ROC-space is considered to be better than another if it corresponds to a higher sensitivity and specificity at a time, i.e., if it is closer to the point with coordinates (0, 1), which represented a perfect classification case (100% sensitivity and 100% specificity). Informally speaking, one model is better than another if most of the corresponding curve is closer to point (0, 1) than most of the curve corresponding to the second model.

## 3. Results and Discussion

### 3.1. Spectral Analysis

**Figure 1:**
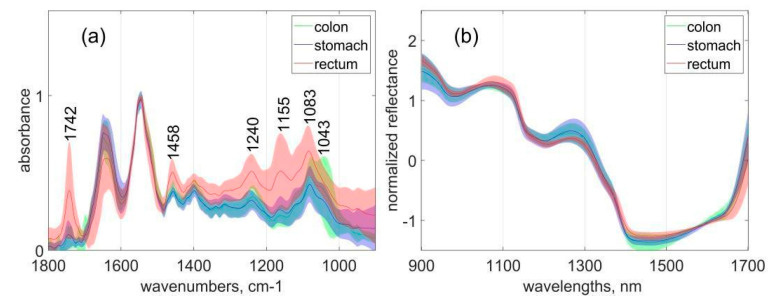
The mean spectra and the intervals of standard deviation for normal colon, stomach, and rectum samples: (**a**) unpreprocessed mid-infrared (MIR) spectra; (**b**) smoothed standard normal variate correction (SNV)-normalized near-infrared (NIR) spectra. The curves and the surrounding coloured regions represent the mean spectra and the standard deviation intervals of the respective data variables.

Mid-infrared absorption and near-infrared reflection spectra were collected from stomach, colon, and rectum tissues, including malignant and normal species. In particular, 52 measured positions of colon samples, 52 measured positions of stomach samples, and 21 measured positions of rectum samples were selected to be included in data analysis based on the spectral quality of the raw data. From each study subject, the average spectra of the normal and tumour tissues were calculated. NIR and MIR spectra of normal tissue samples from colon (green), stomach (blue), and rectum (red) are presented in Figure 1a,b correspondingly.

Significant differences between organs were observed in the whole spectral interval for both methods, MIR and NIR spectra. The overall intensity distribution of MIR absorption was much higher for rectal tissue in comparison with colon and stomach tissues. In the rectal tissue, spectral differences were especially pronounced in the region of 1800–1700 cm^−1^, which can be assigned to the carbonyl stretching vibration of lipids at 1742 cm^−1^, and in the correlating interval surrounding 1458 cm^−1^, which can be associated with lipid CH2 bending or scissoring vibrations [45,46,47]. Spectral differences for rectal tissue were also observed at around 1240 cm^−1^ caused by the asymmetric stretching vibrations of PO_2_ associated with phosphate and a nearby peak at 1155 cm^−1^ that can be assigned to the C–OH stretching mode by amino acids (phenylalanine, threonine, tyrosine, serine) from cell proteins [48]. An additional pronounced spectral difference of the rectal tissue is related to the presence of a peak at 1083 cm^−1^. This peak is associated with symmetric stretching vibrations of the PO_2_ group that mainly originate from phospholipids and represents an increased concentration of cellular nucleic acids [49]. In the colon tissue, an additional spectral peak was observed at 1043 cm^−1^, which is assigned to the C–O stretching band coupled with C–O banding of C–OH groups of glycogen [50]. Differences in the NIR reflection spectra cannot be interpreted as straightforward as MIR spectra because of the absorption peaks broadness (up to 100 nm) and overlapping. However, several intervals can be highlighted, including the CH stretching second overtone (≈1100–1200 nm) and CH combinations first overtone of OH and NH bonds (≈1300–1700 nm) [51]. Significant spectral variations related to the specific tissue types described above cause additional limitations regarding the development of a common model based on the full datasets of all three organs. In this case, the best division of the dataset should be considered carefully to find optimal models in the data analysis process. (see Section 3.2).

**Figure 2:**
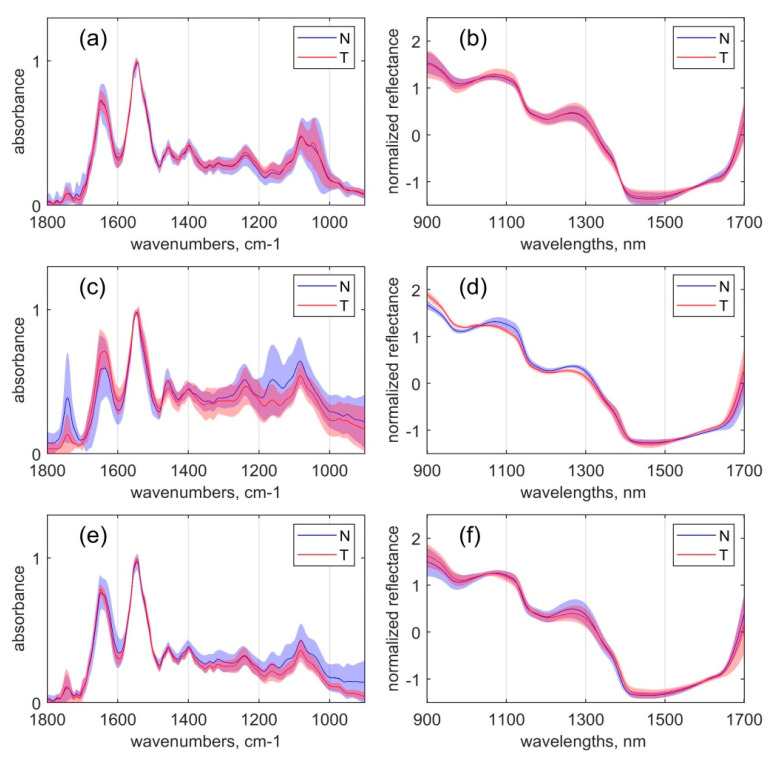
The mean spectra and the standard deviation intervals of tumour (designated as T) and benign (designated as N) samples: (**a**) unpreprocessed mid-infrared (MIR) spectra of colon samples; (**b)** smoothed SNV-normalized near-infrared (NIR) spectra of colon samples; (**c**) unpreprocessed mid-infrared (MIR) spectra of rectum samples; (**d**) smoothed SNV-normalized near-infrared (NIR) spectra of rectum samples; (**e**) unpreprocessed mid-infrared (MIR) spectra of stomach samples; (**f**) smoothed SNV-normalized near-infrared (NIR) spectra of stomach samples. The curves and the surrounding coloured regions represent the mean spectra and the standard deviation intervals of the respective data variables.

As the next step, the alterations between the overall intensity distribution and the spectral shape between malignant and normal tissues were analysed for each organ separately for MIR absorption (Figure 2a,c,e) and NIR reflection (Figure 2b,d,f).

In the colon tissue samples, the presence of a glycogen peak with an average intensity maximum at 1043 cm^−1^ (Figure 2a) caused by symmetric stretching vibrations of the C–O and C–OH groups represents the most significant MIR spectral difference between normal and cancerous tissue [33,52]. Even if the average intensity of the glycogen peak is similar in cancer and normal tissues, the shape of the peak is more distinct in the cancer samples. This finding correlates with other studies that reported higher glycogen concentrations in cancer samples compared to normal samples in both rectum and colon tissues. The increase of glycogen correlates with an increased proliferation rate of tumour cells resulting in a higher degree of glycolysis to meet the cells’ increased energy demand [50,52].

The most significant differences in the NIR spectra of the colon tissue samples were observed at CH first overtone combinations (1400–1600 nm and 1300–1420 nm), which were slightly more pronounced for the tumour tissues [53].

In the rectal tissues samples, the malignant tissue when compared to normal tissue samples showed increased MIR bands at positions of 1640 cm^−1^ and 1550 cm^−1^ associated with amide groups related to proteins. Confirming the results of recent studies [47,54], decreased peaks related to lipids were found at 1742 cm^−1^ and 1400 cm^−1^, and those related to carbohydrates were found at 1160 cm^−1^. The increase in the amide I band (1642 cm^−1^) and amide II band (1550 cm^−1^) can be related to changes in the relative amounts of proteins in the cancer cells during cancer progression [55] and potentially be caused by the unregulated production of cell cytoplasm contents. The decreased peak intensity of the C=O band (1742 cm^−1^) in the cancer sample spectra can be correlated with a higher energy demand during cell proliferation that results in the metabolisation of fats [55].

Regarding NIR spectra, the most significant differences in the rectal tissue samples were observed between 1000 and 1330 nm, corresponding to the CH stretching second overtone region associated with glycoproteins, glycolipids, and carbohydrates [14].

In the gastric tissue samples, the most noticeable distinction of cancer tissue compared to normal tissue samples was the relatively lower bands intensity (representing, therefore, lower content of assigned material) in the spectral region from 1200 to 900 cm^−1^, representing mostly the C–O stretching absorptions from glycogen constituents featured at 1125, 1080, and 1040 cm^−1^ bands. The same trend in stomach tissues was observed by Park et al. and Lee et al. [56,57]. In this study, the lower concentration of glycogen in the stomach tissue of both malignant and normal samples could be ascribed to the application of neoadjuvant chemotherapy to all study participants. Through this therapeutic approach, not only cancer cells but also normal stomach cells could have been inhibited in their cell proliferation. It is reasonable to assume that the healthy stomach lining is similarly affected by chemotherapy, as are the cancer cells due to its naturally rapid proliferation. To confirm this difference, further investigations comparing patients treated and untreated neoadjuvant chemotherapy should be performed. Unfortunately, this is difficult to implement because the application of neoadjuvant chemotherapy is the recommended standard procedure for patients with stomach cancer. Therefore, access to samples of untreated patients is limited.

Spectral differences in the NIR region are most pronounced at the spectral region of OH and NH first overtone (1300–1420 nm).

FTIR spectra of water are comprised of three prominent bands: ~3400, 2125, and 1645 cm^−1^. In our experiment, due to the measurement range, only the third (1645 cm^−1^) H-O-H bending vibration band was overlapping with the vibrations of other chemical compounds. This band should be removed because it can interfere with the bands of other chemical components typically found in biological specimens. The popular way of erasing overlapping water peaks, in studies investigating biological fluids, is the subtraction of water/aqueous solution spectra from the spectra of the samples [58,59]. An artefact that can occur during such a procedure is a negative band in the final spectrum; this is only done when the water band intensity is higher than the intensity of the correlating peak in the sample. We are sure that no over-subtraction of the water band took place during the experiment because the overall absorbance of each tissue sample spectrum was much higher than the overall absorbance of the background (saline solution). The intensity of the reflected light beam in the water solution was lower than in the tissue sample. Nevertheless, an under-subtraction of the water band can occur. To this date, there is no universal and repeatable way of completely erasing this band experimentally. However, to ensure that water content was constant, the surface of each sample was washed with saline (NaCl 0.9%) before the measurement. Even if the subtraction does not completely erase the water signal from the spectra, the impact of that band is drastically decreased. Therefore, the influence of the water absorption on the final model performance is significantly reduced.

Due to the strong, broad absorptions of the water band located at 1645 cm^−1^, there is some degree of overlap, particularly of the amide I peak [60]. Thus, the accurate estimation of amides based on a peak around 1645 cm^−1^ is not always possible. Subtraction of the water band is usually not done in studies investigating tissue samples [60]. Therefore, a peak around 1645 cm^−1^ is mostly higher than the amide II band (1555 cm^−1^). In our study, the amide I peak intensity was decreased by the water spectrum subtraction, and its intensity was lower than the intensity of the amide II band.

### 3.2. Multivariate Analysis

When considering complex datasets including spectra of multiple different organs, an efficient data analysis can be based on a full dataset (one common model) or its subsets comprising spectra of selected organs (separate models). Tissues from different organs have different chemical compositions, which are reflected in the spectroscopic data. The difference in spectra of different organs was investigated by Kondepati et al. and Dybas et al. [51,61], and the difference in the spectra of different tissue types was studied by Barroso et al. and Ralbovsky and Lednev [62,63]. Therefore, each approach to the multispectral analysis (MIR, NIR, combination) was represented by datasets including measurements of one joint or several individual sample sets (colon, stomach, rectum sample sets as well as the full set including all of them), which resulted 12 possible datasets in total.

Due to the manual contact of the probe with the sample surface, there are always minor variations of the contact pressure during a measurement. These kinds of variations can influence the concentration of water, hemoglobin, and lipids. Therefore, even the same kind of samples can have different overall absorbance due to variance in the contact pressure. Reduced contact pressure results in higher diffuse reflectance and scattering [64]. However, all these variations should not influence the spectral ratio intensity between various peaks in the individual spectroscopic signal. To reduce scattering and differences in the overall absorbance between different samples, scattering correction preprocessing methods are usually implemented. In our study, a combination of scattering correction preprocessing was applied to reduce the influence of the contact pressure variations. Every single dataset was tested using different preprocessing methods before PLS-DA modelling: no preprocessing, SNV, second derivative (2D), first derivative (1D), 2D + SNV (2D followed by SNV), 1D + SNV (1D followed by SNV). This combinatorial diversity represents a particular challenge for finding the most efficient modelling strategy.

Concatenated spectral data representing a combination of two spectroscopic methods were tested by independently preprocessing both parts of the concatenated dataset with the same methods as for the individual datasets. Both parts were normalised between 0 and 1, as described in Section 2.5 above. Models with maximal accuracy were chosen to represent single methods (MIR, NIR) or combinations of methods for each sample set. Single method models with the same preprocessings as was used for the individual parts of the concatenated spectral data (combining two spectroscopic methods) are provided in Table 2 (models marked with *). The model loadings are displayed in the Supplementary Materials accompanying the paper. ROC-curves were used to exclude subjectivity of the threshold choice method during model comparison (threshold = 0.5 was used for all models in Table 2.

Two types of cross-validation (CV) were used to validate the models—Standard LOO CV and Monte Carlo CV (see Table 2). These two cross-validation approaches differ in the size of the segment chosen in each iteration of the testing cycle: one position in the case of LOO and 10% of the randomly chosen number of the total number of positions in Monte Carlo. The more commonly used leave-one-out design resulted in more optimistic prediction statistics, while the results of Monte Carlo CV are more reliable because a larger proportion of the data is allocated to the testing set than to the training set when compared to LOO CV. However, both methods worked very similarly in this case for the purpose of comparison between different methods and data blocks, as well as the calibration of data-based prediction.

In general, the validation strategy used in the manuscript is dictated by the necessity to work with a very limited (for ethical and other reasons) dataset. The limited availability of patient samples is one of the main challenges in clinical studies such as this. Nevertheless, we consider this approach, using a two-step validation, sufficient to reach the goals of the work: to investigate the influence of NIR and MIR data combination on the model accuracy (the comparison of different methods of measurement and data analysis), to show the presence of differences between healthy and cancerous tissues of different organs, and to incentivize further efforts aimed at the development of combination methods.

The testing of different preprocessing methods resulted in the selection of the same procedures (either 2D or SNV or their combination) for all datasets. It can be explained by their cumulative effect on the spectra: using derivatives removed the linear variations of the baseline, whereas SNV eliminates the variability of the overall spectral intensity that could be related to the experimental factors, e.g., to the difference of the effective measurement spot or volume.

It can be seen from Figure 1 and Figure 2 that the shapes of MIR and NIR spectra are different for the three considered organs as well as between normal and tumour samples. As such, a non-homogeneity in the data is present; it adds extra complexity to the PLS-DA models (Table 2), which leads to a larger number of latent variables. Kukreti et al. [65] proposed applying particular data preprocessing for analysis that excludes a variation in data associated with the specific sample. In our previous research [33,35], the importance of the choice of preprocessing and the importance of correctly combining different parts of data was also noted.

**Figure 3:**
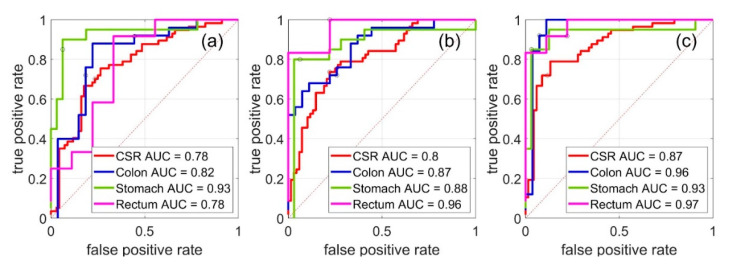
Receiver operating characteristic curve (ROC-curve) of prediction of Leave-one-out (LOO) cross-validation (CV) for models from Table 2. (**a**) Models #1, 4, 7, 10 (models built using NIR data); (**b**) models #2, 5, 8, 11 (models built using MIR data); (**c**) models #3, 6, 9, 12 (models built using concatenated data).

As shown recently [33], the simultaneous use of data obtained by two different spectroscopic techniques can increase the classification accuracy. A similar effect was observed in all our models presented here (see models 3, 6, 9, and 12 in Table 2 and Figure 3). It was also noted that not any pair of methods results in a significant increase in accuracy compared to using these methods separately [33]. In this work, combining two techniques made it possible to obtain an increase in accuracy by 0–15% (calibration and LOO cross-validation accuracy are considered) both on the full set and on subsets, including measurements of individual organs (model #3 was compared with #1,2; #6 with #4,5; #9 with #7,8 and #12 with #10,11). The synergy effect described above is presented in Figure 3. The curves corresponding to models built using concatenated data (Figure 3c) are, in a majority of cases, closer to (0,1) than curves in Figure 3a,b corresponding to models built using single method data. It is important to note that the presented results were obtained from a limited number of samples, not covering all possible variations in the data, which are related to both natural and disease-related differences. Although the obtained research models can be inaccurate for new, undiagnosed clinical samples, the available samples and datasets are well suited for the purpose of comparing the performances of the suggested approaches to the joint data analysis.

Different divisions of the dataset were tested as well. In this case, using a separate model for each respective organ type should be used instead of the joint model. For example, a model built using the colon dataset was used for the discrimination of colon sample measurements etc. This “local” model building approach increased the accuracy (see % acc column in Table 2) by 3–16% for NIR (model #1 was compared with #4,7,10), 0–18% for MIR (model #2 was compared with #5,8,11), and 5–10% for the combination of methods (model #3 was compared with #6,9,12). The same trend is presented in Figure 3a–c. The models built using single organ samples sets (blue, green, and pink lines) were better than the “global” model obtained using the full set of samples (red line; except for the rectum model in Figure 3a, whose corresponding dataset is more limited than the others).

**Figure 4:**
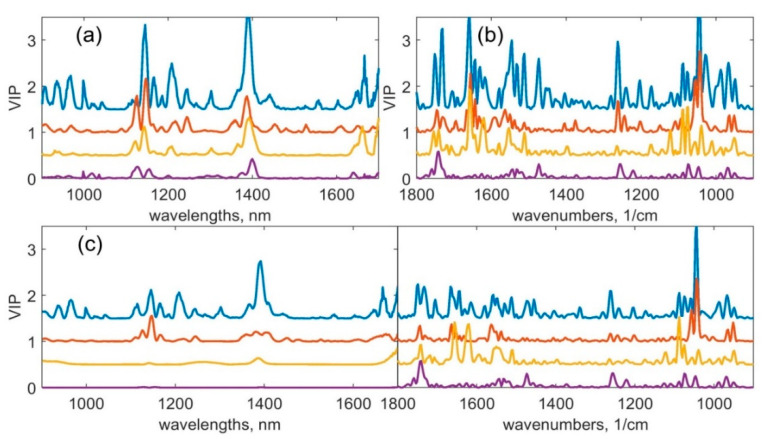
Variable importance in Projection (VIP) for models from Table 2. (**a**) Models #1, 4, 7, 10 (models built using NIR data); (**b**) models #2, 5, 8, 11 (models built using MIR data); (**c**) models #3, 6, 9, 12 (models built using concatenated data). Each curve was shifted up by 30% from the previous curve on the *y*-axis for better visualization of the VIP values. Models built using full datasets (colon, stomach, rectum: CSR) are shown in blue, red—colon datasets, yellow—stomach datasets and violet—rectum datasets.

**Figure 5:**
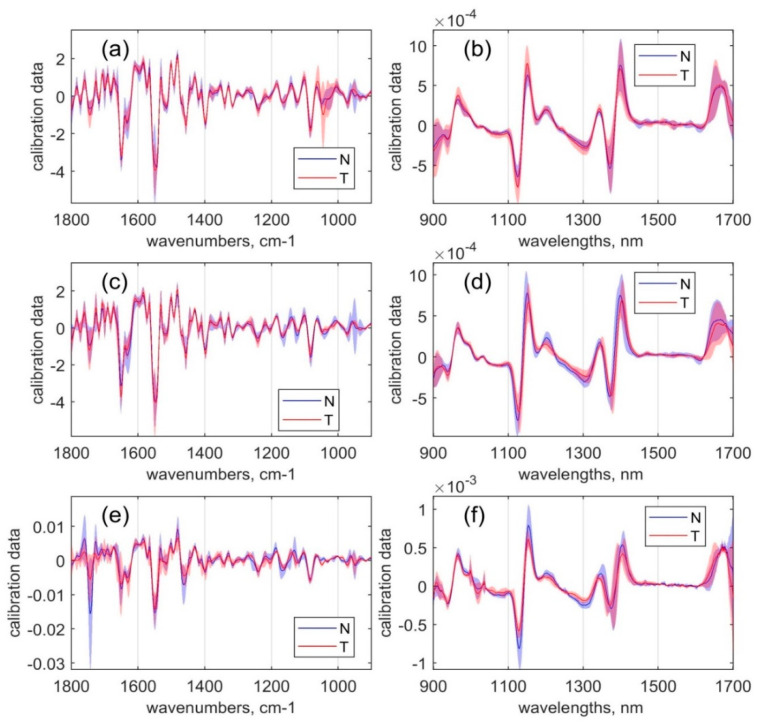
Calibration data (CD) of models built using single methods (before removing the mean centre): (**a**) CD of model #5—MIR dataset pretreated by 2D, SNV including only colon measurements; (**b**) CD of model #4—NIR dataset pretreated by 2D including only colon measurements; (**c**) CD of model #8—MIR dataset pretreated by 2D, SNV including only stomach measurements; (**d**) CD of model #7—NIR dataset pretreated by 2D including only stomach measurements; (**e**) CD of model #11—MIR dataset pretreated by 2D including only rectum measurements; (**f**) CD of model #10—NIR dataset pretreated by 2D including only rectum measurements.

There are many peaks in the VIP values (see Figure 4) because derivative preprocessing for the data was used. Each peak in the original data was recalculated resulting in several less broad peaks by using the derivative. In Figure 5, we displayed the calibration data for models built using separate methods with 2D preprocessing (calibration data for other models are displayed in the Supplementary Materials accompanying the paper). It can be seen from Figure 4 that the intervals harbouring the most pronounced values are different for all models built using NIR data (Figure 4a). This includes models built using concatenated data (Figure 4c, left part), MIR data (Figure 4b), as well as combination models (Figure 4c, right part). Despite differences in VIP values, there are several common intervals: around 1742 cm^−1^, 1650 cm^−1^, and 1043 cm^−1^ in all models built using MIR data and 900–1000 nm, 1100–1200 nm, and 1300–1500 nm in all models built using NIR data. These findings are equal to the differences between normal and tumour tissue measurements described in Section 3.1. Regarding models that were built using concatenated data from rectum samples, the VIP values for NIR spectra (Figure 4c, left part) are less pronounced than the values for MIR spectra (Figure 4c, right part). There is no significant difference between the calibration statistics (see Table 2) for this organ built using only MIR or concatenated data. However, there are differences in LOO CV and Monte Carlo CV, which indicates that using both validation methods results in more accurate conclusions on the presence of synergies when both spectroscopic techniques are combined.

The observed synergy effect is a result of using complementary information brought by two physically different methods of spectroscopy. Evidently, a combination of the complementary information about the tissue properties collected from two different methods and combined in a single modelling approach led to an increase in accuracy of the multivariate discrimination models. In spite of the gain from merging two optical spectroscopic techniques observed in the presented research, an attempt to develop a joint discrimination model comprising several abdominal cancers, i.e., stomach, colon, and rectal cancer, was found to be non-optimal for diagnostics in the presented case. Figure 3 and Table 2 demonstrate that the accuracy obtained using three separate models, one for each specific organ, is generally higher than the accuracy of the joint “global” model for all investigated organs. This can be explained by the major spectral differences observed for the investigated organs (Figure 1). Thus, for further medical applications, it is worth considering the strategy of multiple models rather than using one common model for all abdominal cancer types.

## 4. Conclusions and Outlook

The main challenge for the development of multivariate models that could be incorporated into a medical device lies in the investigation of their applicability limits. As a preliminary step of this development, we compared models built on different sets of samples, including three separate organs: colon, rectum and stomach, which are physiologically related. We demonstrated that the separate models, which were trained to work with samples of one specific organ, are more accurate than the “one common model”, which was trained to work with all considered organs. This statement can be explained by the strong spectral differences observed for all three organs.

Considering possible future applications, a device including a combination of two selected spectroscopic techniques will be able to operate in at least three modes. Based on the practical application, those modes are comprised of (a) a detailed examination of the sample surface (based on MIR direct spectral information), (b) a general in-depth tissue examination (based on NIR spectra), and (c) a more advanced and comprehensive examination (based on concatenated MIR and NIR spectra). The practical benefit of the detailed mode (a) capable of bringing information on the molecular structures on a single-cell scale is the explicit justification of the classification result, which is an essential feature of any medical device. This approach will be especially relevant for hollow organs, such as the ones considered in this study, and it consists of the possibility of non-traumatically analysing tissues before surgery in greater depth.

In addition to the reported improvements in the methods diagnostic potential by means of combining MIR and NIR datasets, there are other promising features of the suggested approach that can produce enormous benefits for the clinical application. We assume that one of the known critical drawbacks of NIR spectroscopy, the necessity to perform an outlier detection step prior to the precise spectral analysis [66], could be eliminated by using additional data of the MIR fingerprint region. Empirically validated and accepted models based on MIR measurements will enable the development of additional models for the surface analysis of specific tissue types, which could be used to choose an appropriate NIR model for the subsequent deeper analysis of tissue.

Beyond these technical considerations, many additional questions remain to be addressed in future studies, which would aim to translate a combined fibre-optic cancer detection probe into the clinics. We would like to emphasize that all validation parameters in this study are calculated from a limited sample set. Therefore, the specific values of sensitivity, specificity, and accuracy may not be maintained in a larger sample set. Nonetheless, the approach of combining NIR and MIR should be applied in further studies based on extended datasets, because it was shown that it yields better results than each method individually. Although, differences in tissue properties between ex vivo samples used for the training of chemometric classification models and in vivo tissue could potentially require an additional adjustment of the modelling algorithms in order to obtain sufficient levels of accuracy and diagnostic reliability. These differences could potentially be caused by the higher oxygenation and perfusion levels of in vivo tissues. Furthermore, the requirement for probe sterility in an intraoperative setting, the need to take repeated measurements in fixed positions, and cleaning the tip of the probe after each measurement remain important challenges to clinical integration. Nevertheless, the introduction of optical spectroscopic tools into clinical cancer diagnostics is a steady trend. Scientific investigations by up-to-date spectroscopic techniques and modern methods of data analysis provide new in-depth knowledge for the successful development of this promising approach.

## Figures and Tables

**Table 1:** Samples used for data evaluation.

Organ	Number of Patients	Number of Samples	Cancer in Tumour Sample (Confirmed)	Absence of Cancer in Normal Sample (Confirmed)
Stomach	19	38 (19N, 19T)	9 (T)/19 (T)	15 (N)/19 (N)
Colon	10	20 (10N, 10T)	9 (T)/10 (T)	10 (N)/10 (N)
Rectum	6	12 (6N, 6T)	6 (T)/6 (T)	6 (N)/6 (N)

**Table 2:** Comparison of spectroscopic and data preprocessing methods for cancer diagnostics.

#	Method	LV ^1^	Pre-processing	Calibration ^5^			Cross-Validation (Leave-One-Out)			Cross-Validation (Monte Carlo)		
				%Se ^2^	%Sp ^3^	%Ac ^4^	%Se ^2^	%Sp ^3^	%Ac ^4^	%Se ^2^	%Sp ^3^	%Ac ^4^
	Colon, Stomach, Rectum (CSR) samples set											
1	NIR	5	2D, SNV	84	84	84	70	76	74	64	78	72
2	MIR	5	2D, SNV	82	81	82	74	78	76	70	70	70
3	Combination	5	2D, SNV | 2D, SNV	91	94	93	72	90	82	68	86	78
	Colon samples set											
4	NIR	5	2D	92	89	90	72	81	77	62	74	69
-*		4	2D, SNV	80	81	81	64	74	69	61	75	68
5	MIR	5	2D, SNV	96	96	96	72	78	75	78	83	80
6	Combination	5	2D, SNV | 2D, SNV	100	96	98	92	93	92	84	96	90
	Stomach samples set											
7	NIR	5	2D	90	94	92	85	94	90	84	92	89
-*		5	SNV	75	94	87	60	81	73	61	82	74
8	MIR	5	2D, SNV	90	97	94	80	94	88	78	92	87
9	Combination	5	SNV | 2D, SNV	95	100	98	85	97	92	75	97	88
	Rectum samples set											
10	NIR	5	2D	100	100	100	92	67	81	87	58	75
11	MIR	5	2D	100	100	100	92	78	86	94	81	89
12	Combination	5	2D | 2D	100	100	100	92	89	90	96	83	90

^1^ Number of latent variables, ^2^ Sensitivity, ^3^ Specificity, ^4^ Accuracy, ^5^ Prediction on a dataset used for model calibration, * Additional models.

## Supplementary Materials

The following are available online at https://www.mdpi.com/1424-8220/20/22/6706/s1, Figure S1: Calibration data (CD), including data of the three organs, of models built using single methods (before mean center removing): (a) CD of model# 2 - MIR data set pretreated by 2D, SNV; (b) CD of model# 1 - NIR data set pretreated by 2D, SNV; Figure S2. Calibration data (CD), including data of the three organs, of model #3 built using concatenated data (before mean center removing): (a) MIR part of data set pretreated by 2D, SNV; (b) NIR part of data set pretreated by 2D, SNV. Both parts normalized to [0,1]; Figure S3. Calibration data (CD) of models built using concatenated data (before mean center removing): (a) CD of model# 6 - MIR part of data set pretreated by 2D, SNV including only colon measurements; (b) CD of model# 6 - NIR part of data set pretreated by 2D including only colon measurements; (c) CD of model# 9 - MIR part of data set pretreated by 2D, SNV including only stomach measurements; (d) CD of model# 9 - NIR part of data set pretreated by SNV including only stomach measurements; (e) CD of model# 12 - MIR part of data set pretreated by 2D including only rectum measurements; (f) CD of model# 12 - NIR part of data set pretreated by 2D including only rectum measurements; Figure S4. X-loadings of models from Table 2: (a) model# 1; (b) model# 2; (c) model# 4; (d) model# 5; (e) model# 7; (f) model# 8; (g) model# 10; (h) mode # 11. Red color corresponding 1st lv, light green - 2nd lv, green - 3rd lv, blue - 4th lv, violet - 5th lv. Each curve was shifted up by 30% from the previous curve on the y-axis for better visualization of the loading plots; Figure S5. X-loadings of models from Table 2: (a,b) model# 3; (c,d) model# 6; (e,f) model# 9; (g,h) model# 12; There are two parts of each plot corresponding to the NIR (a,c,e,g) and MIR (b,d,f,h) parts of concatenated data. Red color corresponding 1st lv, light green - 2nd lv, green - 3rd lv, blue - 4th lv, violet - 5th lv. Each curve was shifted up by 30% from the previous curve on the y-axis for better visualization of the loading plots.

## References

[1] Roser M., Ritchie H. (2015). Cancer. https://ourworldindata.org/cancer.

[2] John S., Broggio J. (2019). Cancer Survival in England-Adults Diagnosed. https://www.nuffieldtrust.org.uk/resource/cancer-survival-rates.

[3] Senkus E., Kyriakides S., Ohno S., Penault-Llorca F., Poortmans P., Rutgers E., Zackrisson S., Cardoso F. (2015). Primary breast cancer: ESMO Clinical Practice. Guidelines for diagnosis, treatment and follow-up. Ann. Oncol..

[4] Hijazi Y., Gondal U., Aziz O. (2017). A systematic review of prehabilitation programs in abdominal cancer surgery. Int. J. Surg..

[5] Hiller J.G., Perry N.J., Poulogiannis G., Riedel B., Sloan E.K. (2018). Perioperative events influence cancer recurrence risk after surgery. Nat. Rev. Clin. Oncol..

[6] Krafft C., Dochow S., Latka I., Dietzek B., Popp J. (2012). Diagnosis and screening of cancer tissues by fiber-optic probe Raman spectroscopy. Biomed. Spectrosc. Imaging.

[7] Flusberg B.A., Cocker E.D., Piyawattanametha W., Jung J.C., Cheung E.L.M., Schnitzer M.J. (2005). Fiber-optic fluorescence imaging. Nat. Methods.

[8] Tu Q., Chang C. (2012). Diagnostic applications of Raman spectroscopy. Nanomedicine.

[9] Bird B., Miljković M., Remiszewski S., Akalin A., Kon M., Diem M. (2012). Infrared spectral histopathology (SHP): A novel diagnostic tool for the accurate classification of lung cancer. Lab. Investig..

[10] Brozek-Pluska B., Dziki A., Abramczyk H. (2020). Virtual spectral histopathology of colon cancer - biomedical applications of Raman spectroscopy and imaging. J. Mol. Liq..

[11] Hui R., O’Sullivan M. (2009). Fiber Optic Measurement Techniques.

[12] Arimoto H., Egawa M., Yamada Y. (2005). Depth profile of diffuse reflectance near-infrared spectroscopy for measurement of water content in skin. Skin Res. Technol..

[13] Moreau F., Yang R., Nambiar V., Demchuk A.M., Dunn J.F. (2016). Near-infrared measurements of brain oxygenation in stroke. Neurophotonics.

[14] Kondepati V.R., Heise H.M., Backhaus J. (2008). Recent applications of near-infrared spectroscopy in cancer diagnosis and therapy. Anal. Bioanal. Chem..

[15] Bogomolov A., Zabarylo U., Kirsanov D., Belikova V., Ageev V., Usenov I., Galyanin V., Minet O., Sakharova T., Danielyan G. (2017). Development and Testing of an LED-Based Near-Infrared Sensor for Human Kidney Tumor Diagnostics. Sensors.

[16] Sakudo A. (2016). Near-infrared spectroscopy for medical applications: Current status and future perspectives. Clin. Chim. Acta.

[17] Yi W.-e., Cui D.-s., Li Z., Wu L.-l., Shen A.-g., Hu J.-m. (2013). Gastric cancer differentiation using Fourier transform near-infrared spectroscopy with unsupervised pattern recognition. Spectrochim. Acta Part A Mol. Biomol. Spectrosc..

[18] Kondepati V.R., Keese M., Mueller R. (2007). Bernd Christoph Manegold, Juergen Backhaus. Application of near-infrared spectroscopy for the diagnosis of colorectal cancer in resected human tissue specimens. Vib. Spectrosc..

[19] Ferrari M., Mottola L., Quaresima V. (2004). Principles, Techniques, and Limitations of Near Infrared Spectroscopy. Can. J. Appl. Physiol..

[20] Guardia M.d.l., Guardia M.d.l., Garrigues S. (2013). Vibrational Spectroscopy. Comprehensive Analytical Chemistry.

[21] Morros J., Garrigues S., Guardia M.d.l. (2010). Vibrational spectroscopy provides a green tool for multi-component analysis. TrAC Trends Anal. Chem..

[22] Baker M.J., Trevisan J., Bassan P., Bhargava R., Butler H.J., Dorling K.M., Fielden P.R., Fogarty S.W., Fullwood N.J., Heys K.A. (2014). Using Fourier Transform IR Spectroscopy to Analyze Biological Materials. Nat. Protoc..

[23] Minnes R., Nissinmann M., Maizels Y., Gerlitz G., Katzir A., Raichlin Y. (2017). Using Attenuated Total Reflection–Fourier Transform Infra-Red (ATR-FTIR) spectroscopy to distinguish between melanoma cells with a different metastatic potential. Sci. Rep..

[24] Bunaciu A.A., Fleschin S., Aboul-enein H.Y. (2015). Cancer diagnosis by ftir spectrophotometry. Rev. Roum. Chim..

[25] Li G., Thomson M., Dicarlo E., Xu Y., Nestor B., Bostrom M.P.G., Camacho N.P. (2005). A chemometric analysis for evaluation of early-stage cartilage degradation by infrared fiber-optic probe spectroscopy. Appl. Spectrosc..

[26] Sablinskas V., Velicka M., Pucetaite M., Urboniene V., Ceponkus J., Bandzeviciute R., Jankevicius F., Sakharova T., Bibikova O., Steiner G. (2018). In situ detection of cancerous kidney tissue by means of fiber ATR-FTIR spectroscopy. Imaging Manip. Anal. Biomol. Cells Tissues XVI.

[27] Finlayson D., Rinaldi C., Baker M.J. (2019). Is Infrared Spectroscopy Ready for the Clinic?. Anal. Chem..

[28] Varma V.K., Kajdacsy-Balla A., Akkina S.K., Setty S., Walsh M.J. (2016). A label-free approach by infrared spectroscopic imaging for interrogating the biochemistry of diabetic nephropathy progression. Kidney Int..

[29] Sreedhar H., Carns M., Aren K., Nazeer S.S., Walsh M.J., Varga J. (2020). Label-free spectroscopic imaging of the skin characterizes biochemical changes associated with systemic sclerosis. Vib. Spectrosc..

[30] Ami D., Mereghetti P., Foli A., Tasaki M., Milani P., Nuvolone M., Palladini G., Merlini G., Lavatelli F., Natalello A. (2019). ATR-FTIR Spectroscopy Supported by Multivariate Analysis for the Characterization of Adipose Tissue Aspirates from Patients Affected by Systemic Amyloidosis. Anal. Chem..

[31] Tunnell J.W., Desjardins A.E., Galindo L., Georgakoudi I., McGee S.A., Mirkovic J., Mueller M.G., Nazemi J., Nguyen F.T., Wax A. (2003). Instrumentation for Multi-Modal Spectroscopic Diagnosis of Epithelial Dysplasia. Technol. Cancer Res. Treat..

[32] Volynskaya Z., Haka A.S., Bechtel K.L., Fitzmaurice M., Shenk R., Wang N., Nazemi J., Dasari R.R., Feld M.S. (2008). Diagnosing Breast Cancer Using Diffuse Reflectance Spectroscopy and Intrinsic Fluorescence Spectroscopy. J. Biomed. Opt..

[33] Bogomolov A., Belikova V., Zabarylo U.J., Bibikova O., Usenov I., Sakharova T., Krause H., Minet O., Feliksberger E., Artyushenko V. (2017). Synergy Effect of Combining Fluorescence and Mid Infrared Fiber Spectroscopy for Kidney Tumor Diagnostics. Sensors.

[34] Chang S.K., Mirabal Y.N., Atkinson E.N., Cox D., Malpica A., Follen M., Richards-Kortum R.J. (2005). Combined Reflectance and Fluorescence Spectroscopy for In Vivo Detection of Cervical pre-Cancer. J. Biomed. Opt..

[35] Ehlen L., Zabarylo U.J., Speichinger F., Bogomolov A., Belikova V., Bibikova O., Artyushenko V., Minet O., Beyer K., Kreis M.E. (2019). Synergy of Fluorescence and Near-Infrared Spectroscopy in Detection of Colorectal Cancer. J. Surg. Res..

[36] Pieszczek L., Daszykowski M. (2009). Improvement of recyclable plastic waste detection—A novel strategy for the construction of rigorous classifiers based on the hyperspectral images. Chemom. Intell. Lab. Syst..

[37] Lee L.C., Liong C.Y., Jemain A.A. (2018). Partial least squares-discriminant analysis (PLS-DA) for classification of high-dimensional (HD) data: A review of contemporary practice strategies and knowledge gaps. Analyst.

[38] Andersen C.M., Bro R. (2010). Variable selection in regression—A tutorial. J. Chemom. Spec. Issue Herman Wold Medal Win..

[39] Wong T.-T. (2015). Performance evaluation of classification algorithms by k-fold and leave-one-out cross validation. Pattern Recognit..

[40] Petersen D., Naveed P., Ragheb A., Niedieker D., El-Mashtoly S.F., Brechmann T., Kötting C., Schmiegel W.H., Freier E., Pox C. (2017). Raman fiber-optical method for colon cancer detection: Cross-validation and outlier identification approach. Spectrochim. Acta Part A Mol. Biomol. Spectrosc..

[41] Krakowska B., Custers D., Deconinck E., Daszykowski M. (2016). The Monte Carlo validation framework for the discriminant partial least squares model extended with variable selection methods applied to authenticity studies of Viagra^®^ based on chromatographic impurity profiles. Analyst.

[42] Pieszczek L., Czarnik-Matusewicz H., Daszykowski M. (2018). Identification of ground meat species using near-infrared spectroscopy and class modeling techniques–Aspects of optimization and validation using a one-class classification model. Meat Sci..

[43] Rinnan Å., Berg F., Engelsen S. (2009). Review of the Most Common pre-Processing Techniques for Near-Infrared Spectra. TrAC Trends Anal. Chem..

[44] Fawcett T. (2006). An introduction to ROC analysis. Pattern Recognit. Lett..

[45] Casal H.L., Mantsch H.H. (1984). Polymorphic phase behaviour of phospholipid membranes studied by infrared spectroscopy. Biochim. Biophys. Acta (BBA)-Rev. Biomembr..

[46] Arrondo J.L.R., Goñi F.M. (1998). Infrared studies of protein-induced perturbation of lipids in lipoproteins and membranes. Chem. Phys. Lipids.

[47] Dong L., Sun X., Chao Z., Zhang S., Zheng J., Gurung R., Du J., Shi J., Xu Y., Zhang Y. (2014). Evaluation of FTIR spectroscopy as diagnostic tool for colorectal cancer using spectral analysis. Spectrochim. Acta Part A Mol. Biomol. Spectrosc..

[48] Simonova D., Karamancheva I. (2013). Application of Fourier Transform Infrared Spectroscopy for Tumor Diagnosis. Biotechnol. Biotechnol. Equip..

[49] Talari A.C.S., Martinez M.A.G., Movasaghi Z., Rehman S., Rehman I.U. (2017). Advances in Fourier transform infrared (FTIR) spectroscopy of biological tissues. Appl. Spectrosc. Rev..

[50] Takahashi S., Satomi A., Yano K., Kawase H., Tanimizu T., Tuji Y., Murakami S., Hirayama R. (1999). Estimation of glycogen levels in human colorectal cancer tissue: Relationship with cell cycle and tumor outgrowth. J. Gastroenterol..

[51] Kondepati V.R., Oszinda T., Heise H.M., Luig K., Mueller R., Schroeder O., Keese M., Backhaus J. (2007). CH-overtone regions as diagnostic markers for near-infrared spectroscopic diagnosis of primary cancers in human pancreas and colorectal tissue. Anal. Bioanal. Chem..

[52] Yano K., Sakamoto Y., Hirosawa N., Tonooka S., Katayama H., Kumaido K., Satomi A. (2003). Applications of Fourier transform infrared spectroscopy, Fourier transform infrared microscopy and near-infrared spectroscopy to cancer research. Spectroscopy.

[53] Chen H., Lin Z., Wu H., Wang L., Wu T., Tan C. (2015). Diagnosis of colorectal cancer by near-infrared optical fiber spectroscopy and random forest. Spectrochim. Acta Part A Mol. Biomol. Spectrosc..

[54] Wan Q.-S., Wang T., Zhang K.-H. (2017). Biomedical optical spectroscopy for the early diagnosis of gastrointestinal neoplasms. Tumor Biol..

[55] Li Q., Hao C., Kang X., Zhang J., Sun X., Wang W., Zeng H. (2017). Colorectal Cancer and Colitis Diagnosis Using Fourier Transform Infrared Spectroscopy and an Improved K-Nearest-Neighbour Classifier. Sensors.

[56] Park S.C., Lee S.J., Namkung H., Chung H., Han S.-H., Yoon M.-Y., Park J.-J., Lee J.-H., Oh C.-H., Woo Y.-A. (2007). Feasibility study for diagnosis of stomach adenoma and cancer using IR spectroscopy. Vib. Spectrosc..

[57] Lee S., Kim K., Lee H., Jun C.-H., Chung H., Park J.-J. (2013). Improving the classification accuracy for IR spectroscopic diagnosis of stomach and colon malignancy using non-linear spectral feature extraction methods. Analyst.

[58] Yang H., Yang S., Kong J., Dong A., Yu S. (2015). Obtaining information about protein secondary structures in aqueous solution using Fourier transform IR spectroscopy. Nat. Protoc..

[59] Fabian H., Lasch P., Naumann D. (2005). Analysis of biofluids in aqueous environment based on mid-infrared spectroscopy. J. Biomed. Opt..

[60] Zohdi V., Whelan D.R., Wood B.R., Pearson J.T., Bambery K.R., Black M.J. (2015). Importance of Tissue Preparation Methods in FTIR Micro-Spectroscopical Analysis of Biological Tissues: ‘Traps for New Users’. PLoS ONE.

[61] Dybas J., Marzec K.M., Pacia M.Z., Kochan K., Czamara K., Chrabaszcz K., Staniszewska-Slezak e., Malek K., Baranska M., Kaczor A. (2016). Raman spectroscopy as a sensitive probe of soft tissue composition—Imaging of cross-sections of various organs vs. single spectra of tissue homogenates. TrAC Trends Anal. Chem..

[62] Barroso E.M., Smits R.W.H., Bakker Schut T.C., ten Hove I., Hardillo J.A., Wolvius E.B., Baatenburg de Jong R.J., Koljenović S., Puppels G.J. (2015). Discrimination between Oral Cancer and Healthy Tissue Based on Water Content Determined by Raman Spectroscopy. Anal. Chem..

[63] Ralbovsky N.M., Lednev I.K. (2019). Raman spectroscopy and chemometrics: A potential universal method for diagnosing cancer. Spectrochim. Acta Part A Mol. Biomol. Spectrosc..

[64] Cugmas B., Bürmen M., Bregar M., Pernuš F., Likar B. (2013). Pressure-induced near infrared spectra response as a valuable source of information for soft tissue classification. J. Biomed. Opt..

[65] Kukreti S., Cerussi A., Tromberg B., Gratton E. (2007). Intrinsic tumor biomarkers revealed by novel double-differential spectroscopic analysis of near-infrared spectra. J. Biomed. Opt..

[66] Pasquini C. (2018). Near infrared spectroscopy: A mature analytical technique with new perspectives—A review. Anal. Chim. Acta.

