# Supplementary material for: Synergy Effect of Combined Near and Mid-Infrared Fibre Spectroscopy for Diagnostics of Abdominal Cancer
Source: Sensors (Basel). 2020 Nov 23;20(22):6706. doi: 10.3390/s20226706 (PMC7700420; doi:10.3390/s20226706)
Supplement: Supplementary file 1 [file sensors-20-06706-s001.pdf]

Supplementary material:

The main goal of the article is a comparison of different approaches to the division of full datasets that include data obtained by two different spectroscopic methods. Calibration data and loadings are presented for models shown in Table 2. The calibration data figures (see Figures S1, S2, S3 and Figure 5) show that chosen preprocessing queues made differences in spectra of normal and tumour samples more visible. In the last figures (S4 and S5) we provided loadings for 5 latent variables for each model shown in Table 2.

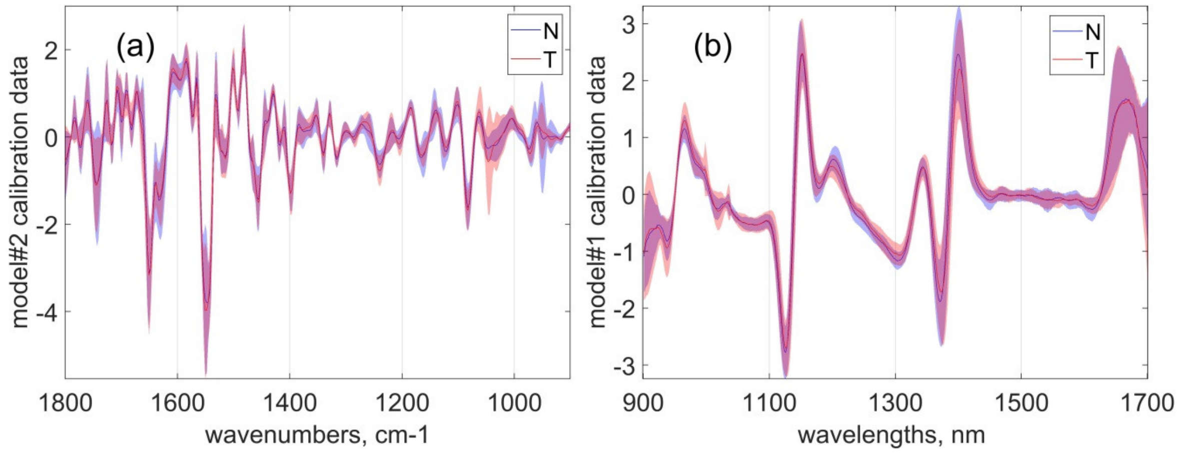

Figure S1. Calibration data (CD), including data of the three organs, of models built using single methods (before mean center removing): (**a**) CD of model# 2 - MIR data set pretreated by 2D, SNV; (**b**) CD of model# 1 - NIR data set pretreated by 2D, SNV.

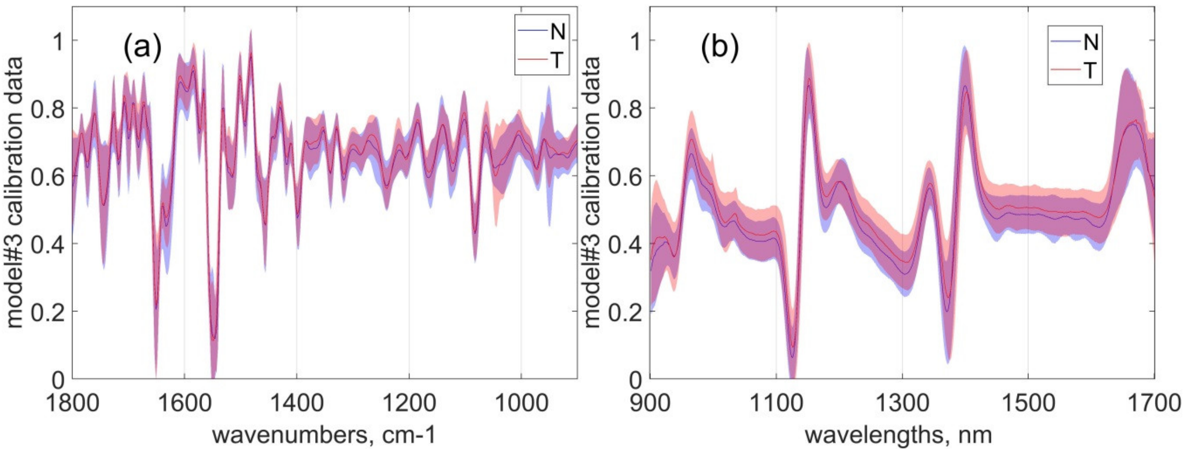

Figure S2. Calibration data (CD), including data of the three organs, of model #3 built using concatenated data (before mean center removing): (**a**) MIR part of data set pretreated by 2D, SNV; (**b**) NIR part of data set pretreated by 2D, SNV. Both parts normalized to [0,1].

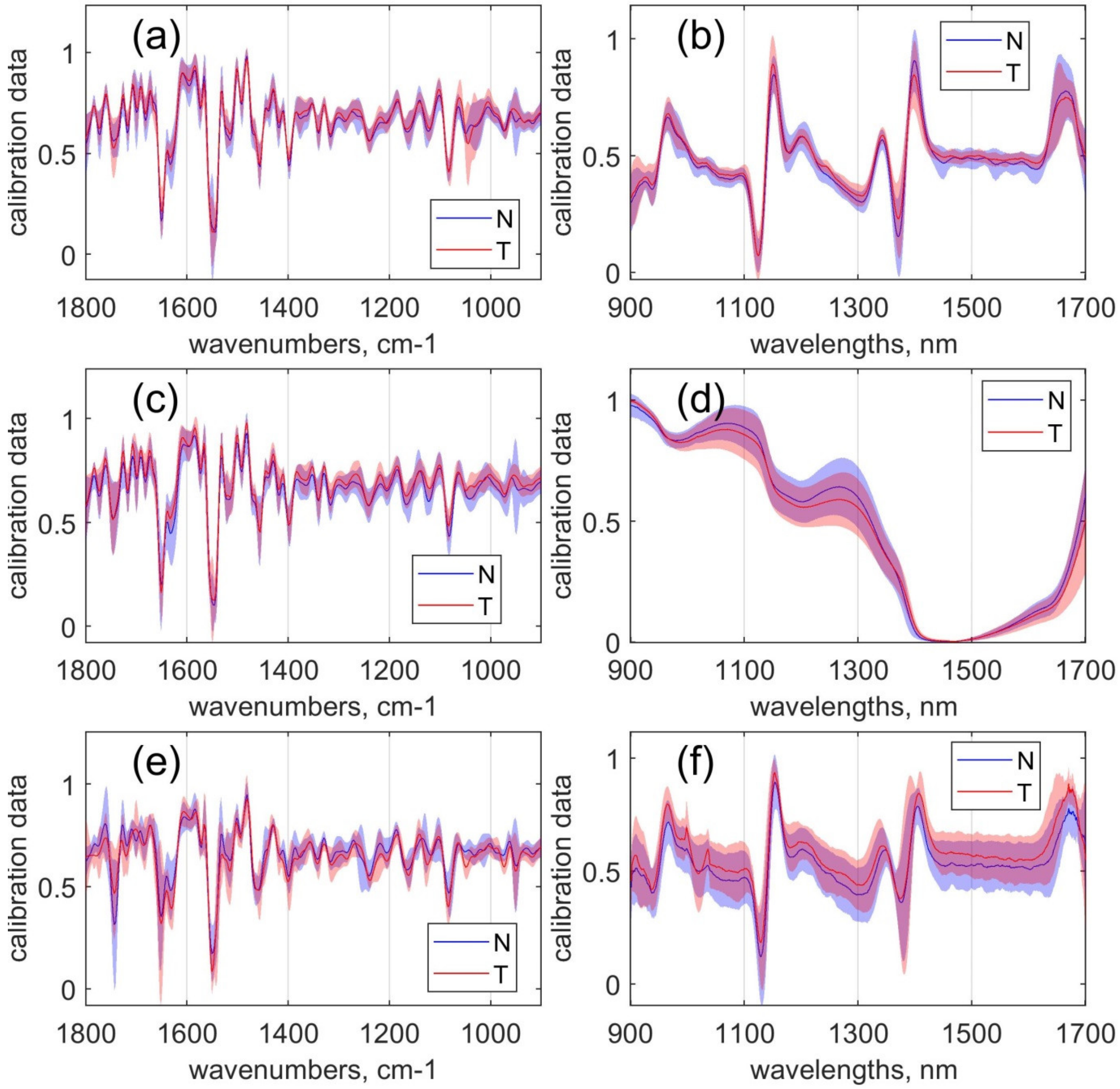

Figure S3. Calibration data (CD) of models built using concatenated data (before mean center removing): (**a**) CD of model# 6 - MIR part of data set pretreated by 2D, SNV including only colon measurements; (**b**) CD of model# 6 - NIR part of data set pretreated by 2D including only colon measurements; (**c**) CD of model# 9 - MIR part of data set pretreated by 2D, SNV including only stomach measurements; (**d**) CD of model# 9 - NIR part of data set pretreated by SNV including only stomach measurements; (**e**) CD of model# 12 - MIR part of data set pretreated by 2D including only rectum measurements; (**f**) CD of model# 12 - NIR part of data set pretreated by 2D including only rectum measurements;

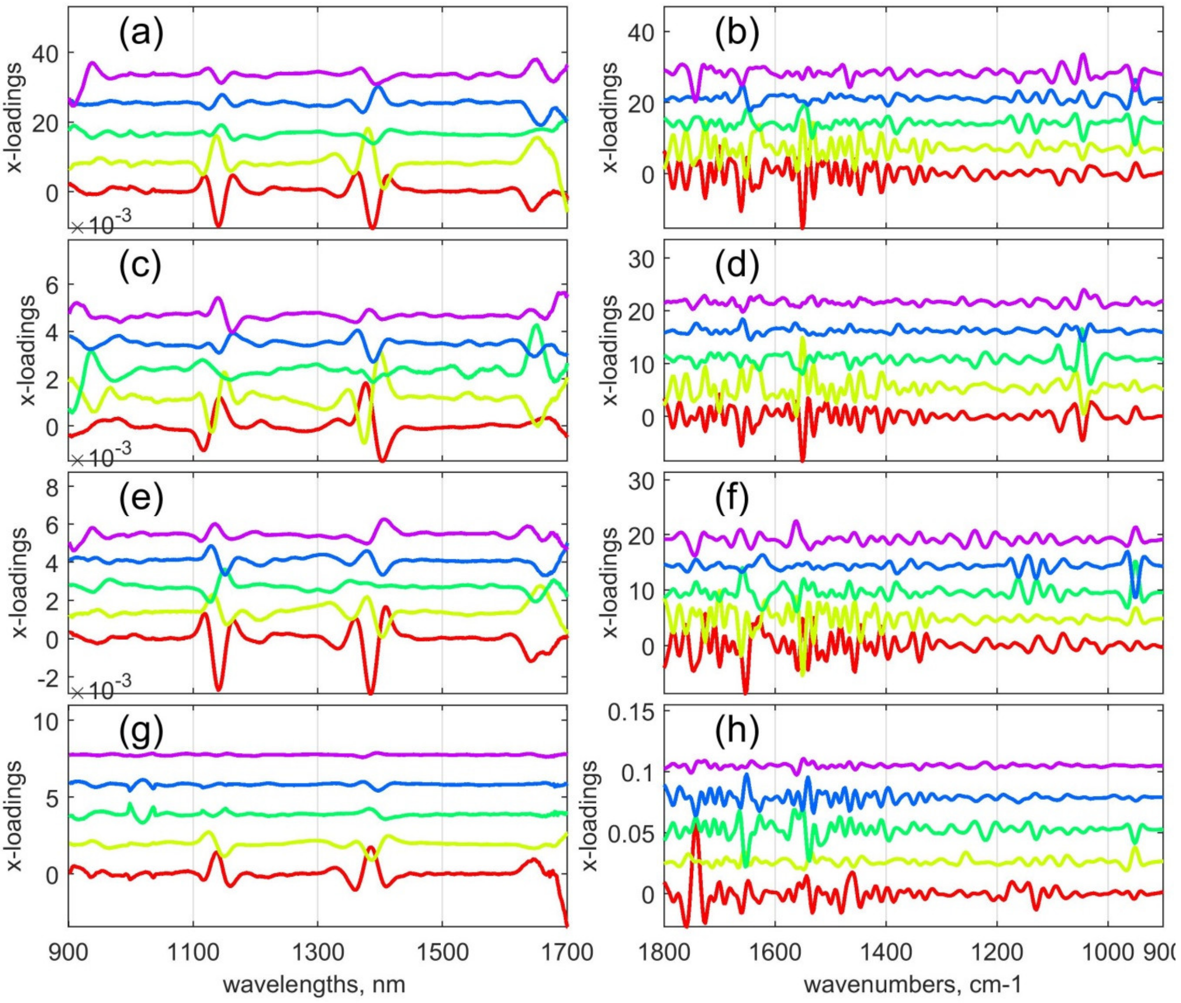

Figure S4. X-loadings of models from Table 2: (**a**) model# 1; (**b**) model# 2; (**c**) model# 4; (**d**) model# 5; (**e**) model# 7; (**f**) model# 8; (**g**) model# 10; (**h**) mode # 11. Red color corresponding 1st lv, light green - 2nd lv, green - 3rd lv, blue - 4th lv, violet - 5th lv. Each curve was shifted up by 30% from the previous curve on the y-axis for better visualization of the loading plots.

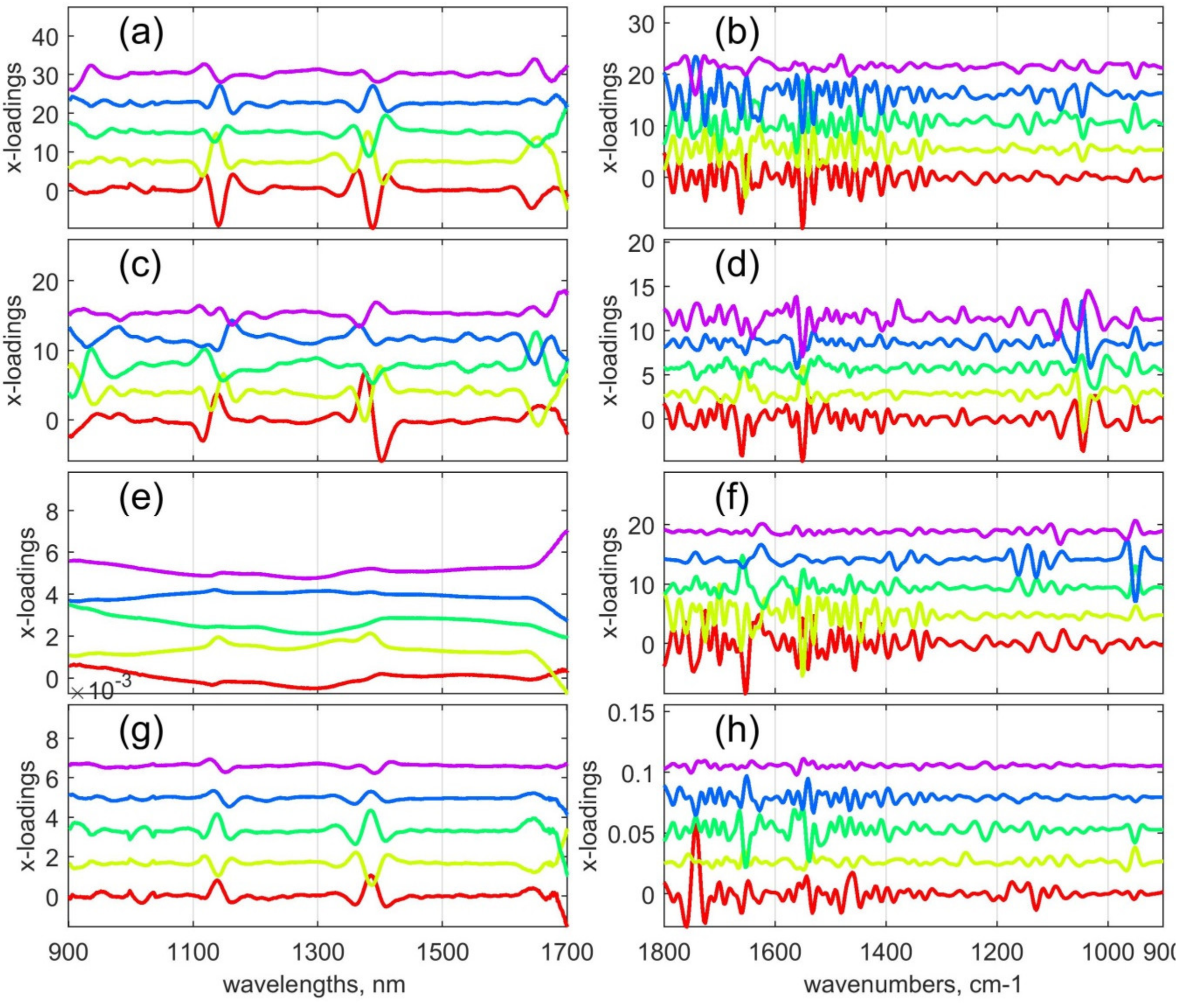

Figure S5. X-loadings of models from Table 2: (**a**,**b**) model# 3; (**c**,**d**) model# 6; (**e**,**f**) model# 9; (**g**,**h**) model# 12; There are two parts of each plot corresponding to the NIR (**a**,**c**,**e**,**g**) and MIR (**b**,**d**,**f**,**h**) parts of concatenated data. Red color corresponding 1st lv, light green - 2nd lv, green - 3rd lv, blue - 4th lv, violet - 5th lv. Each curve was shifted up by 30% from the previous curve on the y-axis for better visualization of the loading plots.
